# Should Digital Interventions for HIV Self-Testing Be Regulated with World Health Organization Prequalification?

**DOI:** 10.2196/60276

**Published:** 2025-08-22

**Authors:** Alex Emilio Fischer, Samanta Tresha Lalla-Edward, Vinodh Edward, Musaed Abrahams, Luke Shankland, John de Wit

**Affiliations:** 1 Department of Interdisciplinary Social Science, Public Health, Utrecht University, Heidelberglaan 1 3584 CS, Utrecht, The Netherlands, 31 302538252; 2 Aviro Health, Cape Town, South Africa; 3 Ezintsha, Faculty of Health Sciences, University of the Witswatersrand, Johannesburg, South Africa; 4 The Aurum Institute, Johannesburg, South Africa

**Keywords:** HIV self-screening, digital health, Software as a Medical Device, WHO, vaccine regulation, vaccination, Africa, accessibility, HIV testing, LMIC, World Health Organization, low- and middle-income countries

## Abstract

HIV self-testing (HIVST) allows people to test for HIV outside traditional health facilities, but this presents challenges around pre- and posttest counseling, reporting results, and linking to care. Digital interventions for HIVST, a type of Software as a Medical Device (SaMD), have been shown to address these challenges, but there is currently no standardized system for regulating or approving these interventions. The World Health Organization Prequalification Program (WHOPQ) is an international regulatory body that approves vaccines, medications, and in vitro diagnostics (IVDs) for low- and middle-income countries that do not have the capacity to do their own approvals. This paper explores whether WHOPQ could be used to prequalify digital interventions for HIVST. Over half the World Health Organization (WHO) member states have national regulatory bodies for medical devices, but low- and middle-income countries, especially in Africa, do not have the capacity to regulate medical devices, let alone SaMD. This gap parallels the gap in vaccine regulation that initially led to the development of WHOPQ, and while sophisticated artificial intelligence (AI)–enabled SaMD are being developed, digital interventions for HIVST could be used as a low-risk test case for prequalification of SaMD. The WHOPQ already has a strong history with HIV; over half the WHOPQ funding is from HIV-related funders and half of all prequalified medicines and IVDs are for treatment and diagnosis of HIV; however, only 2% are manufactured in Africa. If digital interventions for HIVST become prequalified, this could improve interoperability and ensure quality, accelerating their adoption at scale. However, care must be taken to remove barriers for African developers and ensure that prequalification does not delay access to people testing for HIV.

## Introduction

HIV self-testing (HIVST) allows an individual to conveniently learn their HIV status by collecting and testing their own specimen (oral swab or blood drop from a finger prick) in the privacy of their own home [1]. In 2012, the first HIVST became commercially available when the Food and Drug Administration (FDA) approved OraQuick for over-the-counter sales in the United States [2]. Since then, a growing body of evidence has supported using HIVST to complement traditional HIV testing programs toward achieving the Joint United Nations Program on HIV/AIDS (UNAIDS) 95-95-95 goals, where 95% of people living with HIV know their status, 95% of those who know their status are receiving antiretroviral therapy, and 95% of those on antiretroviral therapy are virally suppressed, by 2030 [3]. In 2015, only 2 countries integrated HIVST into their national HIV strategy, but after the World Health Organization (WHO) recommended HIVST in the 2016 Guidelines on HIV Self-Testing and Partner Notification [1], and officially prequalified OraQuick in 2017 [4], the number jumped to over 40 [5]. Five years later, there are almost 100 countries that endorse HIVST in their national HIV strategy, as well as 6 prequalified HIVST kits, including one for just US$1 [6,7]. As HIVST gained global popularity, the shift away from health care facilities was recognized to present some challenges for counseling, reporting results, and linkage to care [8,9]. In response, digital interventions for HIVST have emerged as a novel approach for closing these gaps [10-13]. Like other digital health platforms, digital interventions for HIVST use digital technology (apps, social media, messaging platforms, or the Internet) to assist people through an HIVST journey [14]. These digital interventions have been used for demand generation to promote and distribute HIVSTs, but they are also being used to address the challenges experienced with HIVST. Evidence shows that digital interventions for HIVST can deliver standardized counseling, allow users to report results and link them to appropriate care [13].

Before the COVID-19 pandemic accelerated the familiarization of digital health [15,16], the WHO was already drafting its *Global Strategy on Digital Health 2020‐2025* [14]. Subsequently, in 2022, UNAIDS and WHO published a policy brief on *Virtual Interventions in Response to HIV, Sexually Transmitted Infections and Viral Hepatitis* [17]. These documents reaffirm a strong commitment to digital health interventions, including for HIVST, and introduce guiding principles and implementation frameworks. However, these documents do not outline a plan for regulating or prequalifying digital health interventions, unlike for HIVST kits [4].

Nearly 60% of the WHO member states have national regulatory bodies that regulate medical devices, and many of these countries have started to regulate and approve digital health interventions as a category called Software as a Medical Device (SaMD) [18]. A digital health intervention is considered a SaMD if it provides recommendations for the prevention, diagnosis, or treatment of a disease to health care workers, patients, or caregivers [19]. Most low- and middle-income countries (LMICs) do not have the capacity to regulate SaMD, as they are still trying to develop regulatory processes for traditional medical devices. Of the 15 countries represented in the College of Surgeons of East, Central, and Southern Africa, 7 have no regulatory processes, 7 are currently in development, and only 1, South Africa, has an established regulatory process for medical devices [18]. However, despite the established procedure, SaMD in South Africa does not yet require approval, although digital health interventions are considered a Class C IVD medical device, and the South African Health Products Regulatory Authority has committed to regulating all medical devices in the future [20].

These local regulatory authorities are not yet in a position to regulate digital interventions for HIVSTs, so prequalification at an international level may be more suitable. This viewpoint article provides background on the WHO prequalification process and discusses reasons for and challenges related to the WHO prequalification of digital interventions for HIVST.

## WHO Prequalification

The WHO Prequalification Program (WHOPQ) is an international regulatory body that oversees the quality and safety of various medications and medical devices. While many high-income countries have their own regulatory bodies, such as the FDA in the United States or the Medicines and Healthcare products Regulatory Agency in the United Kingdom [19], LMICs do not have the capacity to ensure the quality of these goods, so the WHOPQ was established to serve these countries by identifying products that the WHO deems fit [21]. There are currently over 1700 products on the WHOPQ list, which are categorized into 4 streams: vaccines, medicines, in vitro diagnostics (IVDs), and vector control [22].

The WHOPQ was launched in 1987 to standardize and scale the WHO’s Expanded Programme on Immunization, which had been used since 1974 to determine whether the vaccines for tuberculosis (TB) (Bacillus Calmette–Guérin [BCG]) and Yellow Fever purchased by United Nations Children’s Fund (UNICEF) met suitable quality, safety, and efficacy standards [21,23]. In 2001, the medicines stream was introduced in response to the need for affordable, quality generic medications for the treatment of HIV, which allowed the WHO to begin prequalifying generic antiretroviral drugs (ARVs). Most of the generic ARVs were produced in India, and many procurement agencies did not have confidence in the oversight provided by the Indian drug regulatory authorities, as there were no originator equivalents from highly regulated countries to compare them with [24].

Over the next decade, the prequalification program expanded to include generic medications for TB and malaria, followed by generics from the essential medicines list for reproductive health, pandemic influenza variants, and diarrhea. In 2013, the medicines stream extended from finished pharmaceutical products to include the prequalification of the active pharmaceutical ingredients used to make medications [25]. The IVD stream was introduced in 2011 for the prequalification of HIV tests and expanded in 2013 to include male circumcision devices. In 2014, the IVD stream was redesigned to include tests for malaria, cholera, viral hepatitis, and syphilis. Subsequently, in 2016, HIVSTs were added to the IVD stream. The vector control stream was also added in 2016 [25].

For a product to become WHO prequalified, it must go through various assessments. The process varies somewhat across streams, but always starts with an invitation for expressions of interest for a specific product category released by the WHO. Manufacturers are invited to submit a cover letter, product dossier, and product samples (including final packaging and instructions for use) to undergo evaluation against internationally accepted regulatory standards for safety, quality, and efficacy [21]. The evaluation also includes a presubmission screening for eligibility and inspections of the manufacturing and clinical trial sites, where applicable. If successful, the entire WHOPQ process can take up to one and a half years [26], costing the manufacturer up to US$31,000 in fees, depending on the product stream [27].

The gap in regulatory processes for SaMD between high- and low-income countries [18] parallels the gap in regulation surrounding vaccines and medications that initially led to the development of the WHOPQ [21]. Since 2017, there have been no new streams added to WHOPQ. However, it has been suggested that the WHOPQ process could be expanded to include digital health technologies in the future [28]. This suggestion corresponds with the proposed actions of the WHO *Global Strategy on Digital Health 2020‐2025*, which includes the development of a WHO framework for assessing and regulating digital health technologies [14].

## Reasons for Prequalification of Digital Interventions for HIVST

### WHOPQ and HIV

The SaMD space is large and diverse, with an estimated 350,000 digital health apps ranging from simple educational chatbots to advanced AI algorithms that use machine learning for early cancer diagnoses [29]. There are approximately 50 digital interventions for HIVST for which a peer-reviewed publication is available [13], and they do not provide a diagnosis but are intended as a screening tool to educate users and link them to care for confirmatory testing and appropriate treatment [10]. Due to their small number and built-in screening-confirmation control, digital interventions for HIVST could provide a low-risk test case for the WHOPQ to develop preliminary prequalification guidelines for SaMD.

The WHOPQ also has a strong history in the domain of HIV. Although the program started with the prequalification of vaccines, expansion into new product streams has been guided by the need to diagnose and treat HIV [23]. In 2002, the first medicine prequalified by the program was Ritonavir, an ARV, and the 10 other medications prequalified in the first year of the WHOPQ were all for treating HIV. A decade later, when the prequalification of IVDs was added to the program, the first prequalified IVD was for malaria, then the following 6 were all for the diagnosis of HIV. Currently, almost half the prequalified medicines (46%; 297/646) are for the treatment of HIV and more than half of the IVDs (57%; 62/109) are for HIV diagnosis [4].

Since 2006, the primary WHOPQ funder has been UNITAID, whose mission is to increase access to treatment for HIV, TB, and malaria, especially in LMICs. In 2017, UNITAID accounted for over half the WHOPQ budget, with supplementary funding from other HIV-related donors, including the Bill and Melinda Gates Foundation and The Global Fund to Fight HIV, TB, and Malaria [4]. With a proven track record of introducing new product streams by prequalifying solutions for HIV treatment and diagnosis, and the majority of WHOPQ funding coming from HIV-related donors, the prequalification of digital interventions for HIVST could be taken up by WHOPQ as a way of exploring the introduction of a new SaMD stream.

### Improved Data Quality and Interoperability

The International Medical Device Regulators Forum (IMDRF) is a voluntary group of national medical device regulators, chaired by the FDA. IMDRF’s objective is to define and standardize medical device registration, and in 2013 they formed the SaMD Working Group [18]. This working group initially defined SaMD using standardized vocabulary and then published a framework for risk categorization, a quality management system, and the clinical evaluation of SaMD [18].

These working group documents are supplemented by guidance from the International Organization for Standardization (ISO) and the International Electrotechnical Commission (IEC), independently producing guidance documents for medical devices and software. ISO 13,485 and ISO 14,971 define the requirements for quality and risk management for SaMD, while ISO 82,304 is directly concerned with health apps. Furthermore, IEC 62,304 and IEC 62,366 address the software lifecycle and usability engineering processes [30,31]. SaMD manufacturers must also consider the Fast Healthcare Interoperability Resources (FHIR) regulations, which outline the ideal rules for collecting and exchanging health care data electronically, and the European Union’s General Data Protection Regulation (GDPR), which ensures data security and privacy [32,33].

Despite this abundance of guidelines and frameworks, not all regulatory bodies adopt and follow them with the same rigor [34]. A study of 9 countries found that most countries had centralized regulatory authorities that use a framework to evaluate health apps [35]. However, these countries had varying oversight of the usability, accountability, and interoperability of the apps [35].

Interoperability is especially important for digital interventions for HIVST, as there is a need to record results for linkage to care and epidemiological tracing. Furthermore, there is a need to standardize how to capture information from emerging populations of HIV testers, including individuals on PrEP and people living with HIV who continue to self-test. However, these indicators are not included or defined in the WHO document recommending HIV self-testing [36]. By prequalifying digital interventions for HIVST, the WHOPQ could consolidate data collection and de-silo interoperability issues.

### Adoption at Scale

Although digital health interventions, including those for HIVST, have shown the ability to improve health outcomes and patient experiences, they are not yet incorporated into traditional budgeting practices that often only consider medications, medical devices, and services [37]. The lack of funding and reimbursement mechanisms makes it difficult for purchasing bodies to select and justify the prioritization of these digital health interventions at scale. By taking an evidence-based approach, some regulatory bodies such as the UK’s National Institute for Health and Care Excellence (NICE) have introduced assessment frameworks specifically for digital health technologies. If an intervention can prove that it is safe and secure, then it may be formally endorsed by NICE. Although an endorsement does not guarantee national adoption, it is valued as a reliable independent assessment by decision-making bodies, when determining which digital health interventions to prioritize for funding [37].

Regarding digital interventions for HIVST, anything that will accelerate their widespread adoption will create a positive impact. With faster access, these promising interventions would guide more people through HIV testing and link more people living with HIV to life-saving ARV treatment. Early access to ARV treatment increases the life expectancy and quality of life of people living with HIV. In addition, there are epidemiological benefits at the population level, as ARV treatment decreases HIV viral load, and when the viral load is undetectable, HIV becomes untransmittable [38]. Furthermore, newly diagnosed people living with HIV change their sexual behavior after learning their status, leading to increased condom use and fewer sexual partners [39].

Many UN agencies, nongovernment organizations (NGOs), and donors, such as the Global Fund, now require WHOPQ for bulk purchasing of medicines and IVDs. Although an endorsement by national regulatory bodies would be beneficial, prequalifying digital interventions for HIVST would likely have a much larger impact on their widespread adoption at scale [40]. The widespread adoption of HIVST has also paved the way for the self-testing of other sexually transmitted infections, such as hepatitis and syphilis [41]. These other sexually transmitted infection self-tests follow the same procedures as HIVST, so digital interventions for HIVST could be easily adapted and applied to promote these tests as well.

## Concerns Regarding Prequalification of Digital Interventions for HIVST

### More Important Digital Health Priorities

The regulation of SaMD is on the WHO roadmap, and while digital interventions for HIVST could offer a low-risk pilot, there may be more impactful digital interventions to start with. In 2019, the WHO held its first Digital Health Technical Advisory Group meeting, which discussed how a prequalification process could be applied to data analytics, specifically the predictive algorithms used in AI-enabled SaMD [28]. It would be challenging to prequalify a learning algorithm that is constantly changing and adapting. However, these algorithms are already in use and, in some cases, have been shown to outperform their human counterparts [42]. AI-enabled SaMD is effective in spotting cancerous tumors through image-based AI algorithms, performing insulin dose calculations for diabetes through AI-enabled decision trees, and predicting disease patterns through algorithms developed during COVID-19 [42,43]. AI is also being piloted in digital interventions for HIVST, with computer vision technology to interpret test results, instead of untrained human users [44].

Despite these benefits, there are also ethical, privacy, and liability risks related to AI-enabled SaMD. An example of an ethical risk is racial bias. One study revealed that in a risk scoring algorithm, Black patients were significantly sicker than their White counterparts with the same score. If race were corrected for in the algorithm, the number of Black people eligible for additional care would increase from 17.7% to 46.5% [45]. In this example, incorrect assumptions in the training data led to bias. In addition, there are also privacy concerns regarding ownership and security of the data used to train the AI models. Furthermore, there are liability implications, especially with emerging algorithms used for diagnosis and treatment in clinical environments, where they may present a substantial risk to people’s health and well-being [46]. Despite these considerable risks, many AI-enabled algorithms are already being trained and used in health care facilities without regulation. Prequalifying these SaMD by the WHOPQ may present a critical opportunity to positively impact patient outcomes.

### Existing Barriers to WHOPQ

With almost 26 million people living with HIV, Africa accounts for over two-thirds of the global disease burden of HIV [47]. Despite such a high disease burden, less than 2% (5/297) of the prequalified medications for HIV were from African manufacturers; Lamivudine/Zidovudine (Universal Corporation Ltd., Kenya and Aspen Pharmacare Ltd.), Lamivudine/Zidovudine+Nevirapine (Aspen Pharmacare Ltd.) and Ceftriaxone, 500 mg and 1000 mg (Egyptian International Pharmaceutical Industries Company). Regarding HIV diagnostics, none of the 62 prequalified IVDs were from African manufacturers; however, one device, the Mylan HIV self-test (Atomo Diagnostics), has a secondary manufacturing site in South Africa [4].

The cost of research and development for medications and IVDs is a crucial barrier to entry that keeps 98% of these items from being manufactured in Africa [4]; however, the obstacles to developing digital interventions are much lower. There are over 7000 tech start-ups in Africa, and in 2021, these African start-ups raised over USD 2 billion [48,49]. Most of these start-ups develop fintech and e-commerce platforms, with less than 6% of the total funding going to digital health start-ups [50]. For these digital health start-ups, the cost of USD 31,000 for WHOPQ may be prohibitive, especially considering that the process could keep their product out of the market for at least one and a half years before the prequalification is completed [24,25].

In 2024, the Africa Centers for Disease Control and Prevention (CDC) launched the Africa HealthTech Marketplace as a way to empower local innovators by showcasing a variety of locally designed digital health solutions [51]. The marketplace is intended to improve access to local digital health interventions by categorizing products and resources from trusted sources, although it does not provide regulatory approvals. While this platform is an important step forward, there is no evidence yet to suggest that NGOs will base purchasing decisions on the marketplace, like they do with WHOPQ.

## Conclusions

Access to the market of SaMD is regulated nationally by more than half of the WHO member states. However, despite the IMDRF, ISO, and EIC frameworks, there is no standardized international regulatory board for digital health interventions. The WHOPQ provides regulatory approvals for vaccines, medication, and IVD, which could also be used to prequalify SaMD. National regulatory bodies, such as NICE in the United Kingdom, can endorse digital health apps, while the CDC’s new Africa HealthTech Marketplace can showcase local innovations; however, neither of these options is as influential as WHOPQ, which many funders now require for bulk purchases.

Digital interventions for HIVST could be considered for a low-risk pilot case, based on the WHOPQ’s history with lifesaving HIV interventions and the need to standardize data and interoperability issues. If prequalification of digital interventions for HIVST were to become available, care should be taken to ensure that the process does not inequitably introduce barriers for digital health companies from LMICs, including in Africa, and that the prequalification does not unnecessarily delay access to people testing for HIV.

## Abbreviations

AI: artificial intelligence
ARV: antiretroviral drug
CDC: Centers for Disease Control and Prevention
FDA: Food and Drug Administration
FHIR: Fast Healthcare Interoperability Resources
GDPR: General Data Protection Regulation
HIVST: HIV self-testing
IEC: International Electrotechnical Commission
IMDRF: International Medical Device Regulators Forum
ISO: International Organization for Standardization
IVD: in vitro diagnostics
NGO: nongovernment organization
NICE: National Institute for Health and Care Excellence
SaMD: Software as a Medical Device
TB: tuberculosis
UNAIDS: the Joint United Nations Programme on HIV/AIDS
UNICEF: United Nations Children’s Fund
WHO: World Health Organization
WHOPQ: World Health Organization Prequalification Program
